# The Role of Attachment and Dyadic Coping in Shaping Relational Intimacy: Actor–Partner Interdependence Model

**DOI:** 10.3390/ijerph192316211

**Published:** 2022-12-04

**Authors:** Anna Wendołowska, Natalia Czyżowska, Dorota Czyżowska

**Affiliations:** 1 Institute of Psychology, Jagiellonian University, 30-060 Kraków, Poland; 2 Institute of Psychology, Pedagogical University of Kraków, 30-084 Kraków, Poland

**Keywords:** attachment, common dyadic coping, relationship satisfaction, intimacy, actor–partner interdependence model

## Abstract

People’s attachment styles play a fundamental role in shaping their intimate relationships. Anxiously attached individuals have a strong need for closeness but a poor ability to obtain the closeness they seek. In contrast, people high in avoidance tend to avoid intimacy in close relationships. Dyadic coping can strengthen the bond between partners, and develop empathy, commitment, sensitivity, and responsiveness to the partner’s needs, which may be the basis of shaping intimacy and closeness. The effects of attachment on relational intimacy were examined, using the actor–partner interdependence model and data from 144 heterosexual couples, aged 26 to 60. Both partners completed measures of attachment, intimacy as a relationship satisfaction dimension, and dyadic coping. The results showed that men’s attachment-related avoidance is related to their own intimacy; also, the avoidant attachment of both spouses is related to each other’s intimacy. The effect of having children on intimacy was significant for men; the effect of financial situation on intimacy was significant for women. Moreover, problem-focused common dyadic coping appeared to have a significant moderating effect on the relationship between attachment-related avoidance and intimacy. Adult attachment and dyadic coping significantly contributed to partners’ relational intimacy.

## 1. Introduction

Establishing and maintaining a close relationship is one of the most important life tasks in adulthood [1,2]. Relationship satisfaction is an important aspect of overall well-being [3,4], while relational dissatisfaction is associated with physical and mental health disorders [5,6], and creates a strong risk factor for relationship breakdown [7,8].

Relationship satisfaction consists of many factors [9]. Major contributions to relationship quality are made by the couple’s intimacy, which is a typical characteristic that partners strive to maintain in long-term relationships [10]. Intimacy is a feature of the developing relationship between people who share their experiences with, and who communicate their thoughts and feelings to, one another. It manifests itself both verbally and non-verbally, and involves many areas of life [11].

Much research has linked the concept of intimacy with terms like “affection” [12], “emotional bond” [13], “closeness” [13], “sexuality” [14], “community” [15], and “sharing” [15]. In the theory of psychosocial development [1], individuals who establish their own identity are able to develop intimacy with another person as a result of a positive solution to a crisis in the period of early adulthood. According to Sternberg [16], intimacy—as one of the three components of love, along with passion and engagement—refers to feelings of closeness, connectedness, and bondedness. Intimacy has both affective and cognitive elements, such as self-disclosure [17], communication of affection [18], perceived partner responsiveness [19], and positive attitudes toward the partner [20].

In our study, intimacy was defined as a high level of satisfaction with the degree of closeness to a partner, openness, trust, and high motivation to work on the relationship [21], all of which is in line with other authors [22], who tend to see intimacy as time spent together, mutual intimate knowledge, mutual support, trust, and affection. The quality of a relationship is the result of a dynamic process, in which partners reveal their personal weaknesses, fears, thoughts, and feelings to each other. The emotional dimension of this process forms the basis of intimacy and closeness [23].

Stress is widely considered to be one of the main elements that reduces relationship satisfaction [24], but it turns out that a moderate level of stress may be beneficial for maintaining positive emotions in a relationship [25]. Stress can reduce or enhance the feeling of positive romantic affection, depending on the intensity of the stressor [26]. How a couple copes with daily stress can have a lasting impact on the relationship [24]. Mutual support in the face of stress can be a specific buffer for negative life experiences, and can shape the perception of a relationship as being full of trust, intimacy, and support [27]. The feeling that both partners are involved in the relationship, and that they can rely on each other, allows both partners to feel connected to each other, and to achieve a high level of intimacy, thereby strengthening their self-esteem [28] and their sense of trust and readiness to help each other [29]. A positive reaction from the partner is important when partners decide to share their problems, and when they want to resolve a conflict or negotiate important personal needs and goals [30]. The basis on which an individual derives resources such as self-esteem or support is called a secure attachment organization [31,32].

An intimate relationship is also affected by attachment patterns [33] that are hypothesized as rational schemas resulting from experience with the caregiver during childhood, which continues to function as a working model for relationships in adulthood [34]. In a secure attachment pattern, the person is confident, and interacts easily with others, meeting both their own and another’s needs; however, when there is a fearful and preoccupied attachment pattern, the person chooses a partner who fits this maladaptive pattern [35,36].

### 1.1. Secure Attachment and Intimacy in a Relationship

Attachment theory coherently explains the formation, development, and dynamics of relationships: its creator, John Bowlby [37], pointed to the critical importance of the bonds formed between child and caregiver, for the child’s further personal and social development. Early attachment relationships are associated with processes of emotional regulation [38]. The caregiver’s accessibility and responsiveness are the basis of the regulatory processes which shape the child’s internal working model, as a mental representation of self, the world, and others; the caregiver’s accessibility and responsiveness are also the basis of developing a secure attachment style [37]. Experiences of rejection, neglect, and inadequate responses from the primary caregiver, to the needs reported by the child, lead to the development of insecure bonds: anxious–ambivalent and avoidant [37]. The attachment system activates in stressful situations, motivating securely attached individuals to seek closeness and support, in order to regulate emotions and manage stress effectively. For people with an avoidant attachment style, closeness is uncomfortable. In a stressful situation, they suppress emotions, and create a physical and mental distance that gives them a sense of autonomy and self-sufficiency [39]. Ambivalent people focus excessively on emotions, and seek immediate support, but this does not lead to emotional calm [32].

The nature of childhood attachment relationships is similar to the romantic relationships we develop in adulthood, and the attachment developed in childhood is the prototype of all close relationships thereafter [37]. Just as in the primary dyad, the caregiver is a safe base and safe haven for the child, so in adult relationships this role is reversible, and in romantic relationships it also includes a sexual element. The emotional bond is represented by experiencing and expressing emotions, the proper expression and regulation of which lead to intimacy [40]. An intimate relationship is often a source of emotions, and allows people to regulate each other’s emotions [41]. A close relationship provides partners with the security and trust that allow them to reveal their most intimate thoughts and feelings, in order to gain understanding and support [23]. The process of building intimacy begins when one partner decides to reveal emotions to the other partner, who may react in an empathetic way by offering support, which strengthens the attitude of emotional exchange. However, if disapproval, contempt or amusement are expressed, the process of building intimacy is disturbed [42]. Especially in conflict situations, a high level of intimacy in the relationship allows partners to show concern or anger, which can initiate the process of solving problems in the relationship.

### 1.2. The Role of Dyadic Coping in Building Intimacy in a Relationship

The process of partners’ mutual emotional regulation occurs through the use of positive and negative dyadic coping strategies [29]. Positive dyadic coping (DC) comes in various forms: (a) supportive DC (focused on emotions and the problem); (b) common DC (undertaking activities together, to solve a problem or reduce negative emotions); and (c) delegated DC (taking responsibility for a partner under stress). Negative DC involves (a) hostile behaviors, such as physically and/or verbally attacking the partner; (b) ambivalent behaviors, such as reluctance to provide support, or lack of conviction in providing support; and (c) superficial behaviors, such as insincere reactions, devoid of empathy [43]. The concept of DC is related to self-perception and to the perceptions of a partner, in the context of communicative competences and behavioral responses to stress. A mutual perception is the result of mutual interaction in the dyad [44], which is also the basis of relationship satisfaction [45]. The literature shows that DC has important effects on relational variables, such as the quality and the harmony of the marriage, and marital satisfaction. DC, both at the general level and at the level of individual strategies of positive and negative DC, is a predictor of satisfaction with the relationship, regardless of gender, age, nationality, level of education, and relationship duration [46]. A high level of positive DC and a low level of negative coping determine the level of satisfaction with a relationship [29,44]. Negative DC is associated with difficulties in regulating emotions [47], and is more often characterized by partners feeling dissatisfied with the level of their relational intimacy [27,48]. Positive DC has a function that is not only related to controlling negative emotions and reducing stress [47]: it also contributes to increasing mutual trust, increasing commitment and intimacy, strengthening relationships, and creating “we-ness” [29].

DC is a feature of well-functioning intimate relationships [49]. People in close relationships are particularly sensitive to the reactions of their partners to stressful situations, as those reactions are important in the context of conflict resolution or offering/receiving support [50]. Open communication, recognition, and adequate responses in a relationship act as beneficial factors in the emotional exchange between partners; they also serve to strengthen the bond [51]. Emotional instability, and an inability to respond adequately to the partner’s needs—both of which characterize anxiously attached people—may lead to a breakdown in interactions [52]. The suppressed emotional reactivity of a partner—which is characteristic of avoidantly attached individuals—may lead to perceiving that partner as unresponsive or insensitive to one’s needs, which translates into a feeling of lack of acceptance and understanding [53], more frequent rumination [54], lower satisfaction [55], and withdrawal from the relationship [56]. Emotional indifference perceived by partners may threaten the functioning of the relationship, and weaken the level of dyadic adaptation [57]. Thus, both constrained and chaotic or unstable emotional dynamics fail to meet the partner’s expectations in a dyadic context. The perception of a partner as non-responsive or inadequately reactive negatively affects relationship satisfaction [58].

A specific form of DC that can be of great importance in relational functioning is common DC, when both partners engage in mutual comfort and emotional regulation [59]. Based on previous research, common DC is the strongest predictor of relationship functioning [60,61], including in the context of attachment [62,63]. Common DC moderates the negative relationship between various aspects of a female’s immigration stress and her relationship satisfaction [64], the relationship between work stress and the quality of marriage [65], and the relationship between stress and verbal aggression [66]. To our best knowledge, no studies have focused on testing the moderating role of problem-focused and emotion-focused common DC in the link between attachment insecurities and intimacy.

### 1.3. The Aim of the Study

The aim of this study was to analyze the association between partners’ attachment, dyadic coping, and intimacy. Both the effect of one’s own attachment and the effect of the partner’s attachment on one’s own and each other’s intimacy were studied.

**Hypothesis 1 (H1)**. *Both women’s and men’s insecure attachment is negatively related to their own intimacy (actor effect).*

**Hypothesis 2 (H2)**. *Both women’s and men’s insecure attachment is negatively related to each other’s intimacy (partner effect).*

We also evaluated whether problem-focused and emotion-focused common DC moderated the actor and partner effects of insecure attachment on relationship intimacy:

**Hypothesis 3 (H3)**. *Problem-focused (H3a) and emotion-focused (H3b) common DC moderate the relationship between women’s and men’s insecure attachment and their intimacy.*

The presented study is part of a larger research project aimed at understanding couple functioning when facing different types of stressor.

## 2. Materials and Methods

### 2.1. Participants

A total of 144 Polish heterosexual couples (*N* = 288) participated in the study. The men were on average 31.9 years old (SD = 9.73) and the women, 30.88 years old (SD = 9.59). Of the men, 44.1%, and of the women, 46.5% had graduated from high school, while 55.6% of the men and 53.5% of the women had at least an undergraduate degree. Most of the men (81.6%) were employed, 18.4% were students, 3.5% were unemployed, and 1.4% were retired; 70.9% of the women were employed, 21.6% were students, 5.6% were unemployed, 1.4% were on maternity leave, and 0.7% were retired. All the couples had been together in a committed relationship for a least a year, and the majority (97.5%) were living together; 64.6% of the couples had no children. Most of the couples (50.3%) had been in their current relationship for 5 years, 22.6% up to 10 years, and 7.6% above 30 years. Only 7.3% of couples assessed their financial situation as bad; 61% said their financial situation was good or very good. The demographic characteristics of the participants are displayed in Table 1.

### 2.2. Procedures

Participants were recruited using traditional methods, such as leaflets, advertisements, and email. The criteria for the selection of respondents were, firstly, that they were currently in a close interpersonal relationship—such as marriage or cohabitation (informal relationship of people living in a shared household)—that had been ongoing for a minimum period of one year, and, secondly, that they were between 25 and 60 years of age. The “minimum-period-of-one-year” criterion was defined in the context of attachment theory [37], which assumes that this is the minimum time sufficient to form a bond between partners. The “age 25–60” criterion referred to the developmental phase of adulthood: the purpose of this criterion was to homogenize the sample as much as possible. All the procedures carried out, being human research, were in line with the ethical standards of the institutional research commission (Ethics Committee of the Institute of Psychology of the Jagiellonian University; KE/01/10/2018) and the Helsinki Declaration of 1964, or comparable ethical standards. Informed consent was obtained from all participants in the study. All participants were remunerated with cinema tickets for their time and effort.

### 2.3. Measures

*The Attachment Styles Questionnaire* (KSP), which was developed by Plopa [21] on the basis of Hazan and Shaver’s [67] concept, consists of 24 statements that make up three scales corresponding to attachment styles: secure (e.g., *I feel good relying on my partner*); anxious–ambivalent (e.g., *It annoys me a lot when I don’t get enough affection and support from my partner*); and avoidant (e.g., *I don’t feel well when my partner tries to be very close to me*). The attachment styles questionnaire is designed to study attachment styles in romantic relationships. The reliability coefficients of the security, ambivalence, and avoidance scales were good (Cronbach’s α = 0.90, α = 0.84, α = 0.80; McDonald’s *ω* = 0.90, 0.84, 0.88, respectively).

*The Matched Marriage Questionnaire* (KDM-2; [68]) examines the level of satisfaction with the relationship (assessed by both partners) in terms of intimacy (e.g., *As the years of marriage go by, our intimacy, trust, and unity increase between us*), self-fulfillment (e.g., *I think marriage is the best way to live, love, and work*), similarity (e.g., *We have the same or similar views on how to raise our children*), and disappointment (e.g., *I treat my spouse as someone who has failed my expectations*). The combined subscale scores give an overall level of satisfaction with the relationship. In the presented study, the intimacy subscale was included in the analysis. The reliability of the scale was good (Cronbach’s α = 0.87, McDonald’s *ω* = 0.80).

*The Dyadic Coping Inventory* (DCI; [69]) validated by Wendołowska et al. [70], is a 37-item questionnaire assessing how couples cope with stress, which consists of five scales assessing dyadic coping (DC) by self and by partner: stress communication (SC, e.g., *I openly tell my partner how I feel and when I need support*); emotion-focused supportive DC (e.g., *I show my partner compassion and understanding*); problem-focused supportive DC (e.g., *I help my partner to see stressful situations in a different light*); delegated DC (e.g., *I take on things that my partner would normally do in order to help him/her out*); and negative DC (e.g., *I do not take my partner’s problems seriously*). Item contents, and the number of items per subscale, are equivalent across self and partner. There are also two scales for common DC: problem-focused common DC (e.g., *We try to cope with the problem together and search for ascertained solutions)* and emotion-focused common DC (e.g., *We help each other relax with things like massage, taking a bath together, or listening to music together*). The respondents complete the inventory by marking their answers on a 5-point scale. The total DC score is the sum of items 1–35 after inverse coding of the negative behavior scale items. Items 36 and 37 are not included in the overall score. A total score of less than 111 means low DC; a total score greater than 145 signifies high DC. The DCI is a widely used self-report instrument designed to measure dyadic coping in couples or individuals currently involved in a romantic relationship. The DCI is also useful for clinical practice and research into relationships, couples, and families, measuring communication and perceptions about the quality and quantity of positive and negative partner support in the face of stress [71]. The scale reliability is α = 0.88 and *ω* = 0.84. In the presented study, two scales for common DC were included in the analysis: problem-focused common DC and emotion-focused common DC.

A *Demographic questionnaire* created by the researchers was used to collect demographic data, including age, gender, length of relationship, type of relationship (formal or informal), number of children, employment status, education level, and financial situation, with response options “poor”, “rather good”, “good”, and “very good”.

### 2.4. Analysis Strategies

The means with standard deviation were calculated for all variables. Spearman’s correlations were used to examine the intercorrelational matrix among the variables; the Wilcoxon signed-rank test for paired samples was used to analyze gender differences in the variables. Correlations for each variable between men and women assumed a lack of interdependence of the results in the dyads [72]. We expected significant correlations between the partner’s attachment, common DC, and relational intimacy. 

**Figure 1:**
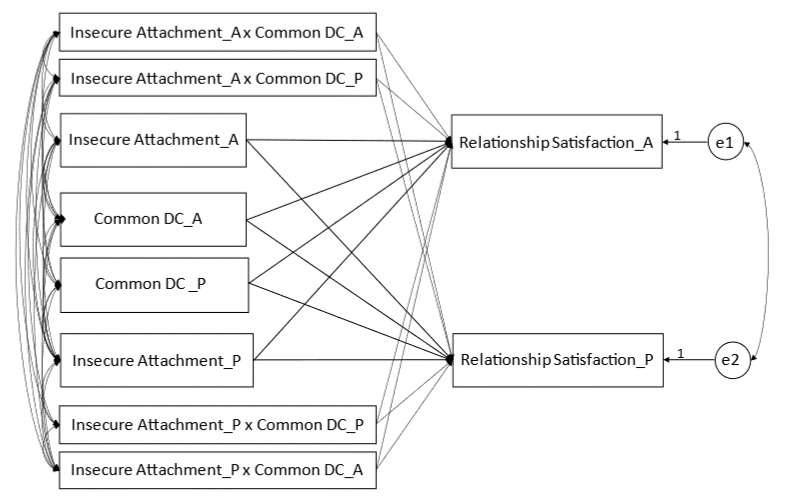
Actor–partner interdependence model, with common DC as moderator. The rectangles represent the independent and dependent variables. The two circles present the latent error terms. The arrows describe the actor and partner effects. × represents interactions of independent variables and moderators of females (_A) and males (_P). The curved double-headed arrows on the left represent the covariances between the independent, moderating, and interaction variables, and the curved double-headed arrow on the right, the correlation between the two error terms.

The variables of both partners were considered as a dyadic construct, so we used the actor–partner interdependence model (APIM) for the analysis [73], which allowed us to assess simultaneously the actor effects (e.g., the association between the female’s attachment and her own intimacy) as well as the partner effects (e.g., the association between the male’s attachment and his own intimacy), while accounting for the within-couple dependency in the data structure [74]. All analyses were performed as part of Structural Equation Modeling (SEM; [75]), with maximum likelihood estimation, using the lavaan package [76]. To test the differences between the genders, the differences between the actor effects in a woman and a man were calculated, as well as the differences between the partner effects in a woman and a man [74]. In the case of the simple APIM (model 1), with insecure attachment as a predictor, and relationship satisfaction as a result, we expected insecure attachment to have significant negative actor (H1) and partner (H2) effects on relationship satisfaction in women and men. In model 2, we estimated insecure attachment’s actor and partner effects on relationship satisfaction, controlling for covariates, such as “length and type of relationship”, “having or not having children”, and “financial situation”. In H3, we proposed moderating the role of common DC in the relationship between insecure attachment and relationship satisfaction, with which the moderator may interact [77]. We analyzed the effect, on relationship satisfaction, of problem-focused (model 3) and emotion-focused (model 4) common DC interaction with insecure attachment, using the actor–partner interdependence moderation model (APIMoM), which is an extension of the APIM [78]. There are two potential moderators of the actor and partner effects in both models (3 and 4): the actor moderator (own common DC) and the partner moderator (partner common DC). Then there are two actor effects and two partner effects of the moderating variable. Using gender as the differentiating variable, eight interaction effects can be potentially analyzed. To estimate these moderating effects, four two-way interactions were added as predictors in the models (Figure 1): female (_A) Insecure Attachment x female (_A) Common DC; female (_A) Insecure Attachment x male (_P) Common DC; male (_P) Insecure Attachment x male (_P) Common DC; and male (_P) Insecure Attachment x female (_A) Common DC. 

We tested the models with the effects constrained to be equal across gender, which reduced the number of possible interaction effects from eight to four: (1) the actor effect moderated by the actor’s moderator (Insecure Attachment_A × Common DC_A → Relationship Satisfaction_A and Insecure Attachment_P x Common DC_P → Relationship Satisfaction_P); (2) the actor effect moderated by the partner’s moderator (Insecure Attachment_A x Common DC_P → Relationship Satisfaction_A and Insecure Attachment_P x Common DC_A → Relationship Satisfaction_P); (3) the partner effect moderated by the actor’s moderator (Insecure Attachment_P x Common DC_A → Relationship Satisfaction_A and Insecure Attachment_A x Common DC_P → Relationship Satisfaction_P); (4) the partner effect moderated by the partner’s moderator (Insecure Attachment_P x Common DC_P → Relationship Satisfaction_A and Insecure Attachment_A x Common DC_A → Relationship Satisfaction_P).

All tests were performed at the significance level of 0.05. A hypothetical model was assessed using chi-square, the root mean square error of approximation (RMSEA; acceptable fit ≤ 0.08), the standardized root mean squared residual (SRMR; acceptable fit < 0.08), the Tucker–Lewis index (TLI; acceptable fit > 0.95), and the comparative fit index (CFI; acceptable fit > 0.95) [79].

## 3. Results

Means, standard deviations, and the Wilcoxon signed-rank test’s probability for paired samples are presented in Table 2. The results of the Wilcoxon signed-rank test, which analyzes the differences between the sexes, indicate that women scored significantly higher than men in terms of intimacy.

The spouses’ results correlated significantly in terms of intimacy, attachment security, attachment avoidance, and common DC (Table 3). In both men and women, a few weak and moderate correlations between the tested variables were observed. For both sexes, security of attachment was linked to the women’s and to the men’s common DC, and to the men’s intimacy. Men’s avoidant attachment correlated negatively with their own and their partner’s intimacy, and their own problem-focused common DC. Significant correlations between the scores of partners on the same variable confirmed the need to control for partners’ interdependence in the APIM.

### 3.1. Avoidant Attachment as a Predictor of Lower Intimacy

The minimum sample size necessary to detect the actor and partner effects for an Actor–Partner Interdependence Model analysis with distinguishable dyads—given a desired level of power (0.80) and alpha (0.05), with beta as a measure of the effect size—is 81 dyads (APIMPower; [80]). Our sample consisted of 144 dyads, so we can conclude that our sample size was sufficient for the APIM analysis. In order to test gender differences, models with distinguishable and with indistinguishable members were compared. The overall test of distinguishability (chi-square(6) = 117.99, *p* < 0.001) confirmed that members of a dyad can be statistically distinguished based on the gender variable [81]. Following Aiken and West’s [82] procedure, the variables were first mean-centered, to avoid multicollinearity.

**Figure 2:**
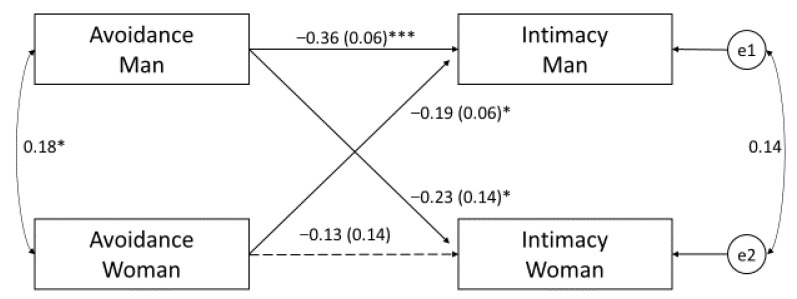
Avoidant attachment and relational intimacy—Model 1. The rectangles represent the independent and dependent variables. The two circles present the latent error terms. The arrows describe the actor (the effect of each individual’s attachment-related avoidance on his or her own intimacy) and partner effects (the effect of each individual’s attachment-related avoidance on his or her partner’s intimacy). The curved double-headed arrows on the left represent the covariances between the independent variables. The curved double-headed arrow on the right represents the correlation between the two error terms. Full lines depict significant paths (* *p* < 0.05; *** *p* < 0.001), and broken lines depict non-significant paths. Standardized coefficients (β), with standard errors in parentheses, are reported.

Although a few correlations were observed between secure and ambivalent attachment styles and partners’ intimacy, no significant actor or partner paths appeared in either women or men in the APIM; therefore, only avoidant attachment was included in further analyses. In model 1, which was the basic APIM (Figure 2), we examined the relationship between avoidant attachment and intimacy. Two hypotheses were tested: (H1), that attachment-related avoidance would predict lower intimacy (actor effects); and (H2), that partners whose spouses were avoidantly attached would experience lower intimacy (partner effects). 

As expected, attachment-related avoidance predicted lower intimacy (H1), but the actor effect was only found in men (Figure 2). A partner’s attachment-related avoidance also predicted lower intimacy in both sexes (H2). There were no significant differences between actor effects (z = 0.33; *p* = 0.74, 95% CI [−0.25, 0.35]) and partner effects (z = −1.70; *p* = 0.09, 95% CI [−0.56, 0.04]), which indicated the same pattern in both genders. The difference in intercepts was 2.36 (z = 0.88; *p* = 0.004, 95% CI [0.75; 3.97]); in the context of predictors, the intraclass correlation between intimacy results for both spouses was 0.14 (*p* = 0.097, [−0.01, 0.12]); therefore, if one partner obtained a high/low score on the intimacy scale as a result of her/his own and the spouse’s attachment-related avoidance, then the other partner also presented a high/low score on the intimacy scale. Model 1 was a saturated model (i.e., df = 0).

In Model 2 (Table 4), in the search for other potential variables that confounded the relationship between avoidant attachment and intimacy, several types of demographic data, including “length and type of relationship”, “having or not having children”, and “financial situation”, were added to the analysis. Power analysis performed at a desired level of power of 0.80 and alpha = 0.05, with beta as a measure of the effect size, indicated 356 observations, which made our sample of 288 slightly too small. Among all the aforementioned potential confounders, “only having children” and “financial situation” appeared to be significant between-dyad covariates. The effect of having children on intimacy was significant, but only for men (β = −1.56; *p* = 0.02). Women with children had a 1.24 lower intimacy score on average, but the effect was not significant (*p* = 0.46). In contrast, the effect of financial situation on intimacy was significant for women (β = 0.95; *p* = 0.036, 95% CI [0.063, 1.836]) but not for men (β = 0.278, *p* = 0.13, 95% CI [−0.078, 0.633]). Despite controlling for the confounding variables, the actor and partner effects for men, and the partner effect for women, still remained statistically significant (Table 4); therefore, it can be concluded that, in the context of the analyzed potential confounding variables, the relationship between partners’ avoidant attachment and intimacy was strong (chi-square(6) = 9.575, *p* = 0.144, RMSEA = 0.065, SRMR = 0.028, CFI = 0.93, TLI = 0.85).

### 3.2. Common Dyadic Coping as a Moderator between Partners’ Attachment-Related Avoidance and Intimacy

It was hypothesized that common DC can moderate the relationship between partner’s avoidant attachment and relational intimacy (H3). We investigated the moderation effect of attachment effects on intimacy, through problem-focused and emotion-focused common DC, using the actor–partner interdependence moderation model. Avoidant attachment, common DC, and intimacy are mixed within-dyad variables, which means that spouses within one dyad may have different scores, and the averaged results of both spouses may also differ between the dyads. 

Before conducting the moderation analysis, the results of the independent variables and moderators were centered (the mean score was subtracted from all the scores). The effects of two models were compared: the model without interaction, and the problem-focused and emotion-focused common DC moderation models (models 3 and 4, respectively). To create a simpler and more interpretable model, indistinguishable dyads with no covariates in both models were allowed, and constraints were placed on interaction effects. 

The goodness-of-fit for model 3 (Table 5), with problem-focused common DC as a moderator, was very good (chi-square(1) = 0.94, *p* = 0.33, RMSEA = 0.00, SRMR = 0.025, CFI = 1.00, TLI = 1.03). In model 3, we observed: (1) statistically significant negative actor and partner effects of avoidant attachment on the partners’ intimacy; (2) the statistically significant actor–partner effect on their own intimacy, of the interaction between a partner’s own attachment avoidance and their spouse’s problem-focused common DC (the term "actor–partner” refers to the interaction between the actor variable of independent variable X and the partner variable of moderator M). The partner’s attachment avoidance effect on their own intimacy weakened as the other partner’s problem-focused common DC increased (partly confirmed H3a). 

The model 4 (Table 5) results show the main significant actor and partner effects of avoidance on intimacy, and the significant actor–actor interaction effect, but the model did not fit the data well (chi-square(4) = 7.97, *p* = 0.093; RMSEA = 0.083, SRMR = 0.079, CFI = 0.73, TLI = 0.78), and so did not support our H3b, that emotion-focused common DC moderates the relationship between avoidant attachment and intimacy. 

## 4. Discussion

The present study sought to better understand intimacy in relation to adults’ attachment and DC. Women’s intimacy correlated negatively with their partner’s avoidance of attachment; men’s intimacy was linked positively with women’s security of attachment, and negatively with own and partner’s avoidance of attachment. People with secure attachment cope better with stress, by identifying the problem and seeking intimacy with others [83]. Contrary to our expectations, no significant relationships were observed between ambivalent attachment and intimacy. This finding was not in line with several studies, where anxious ambivalent individuals were shown to desire heightened levels of closeness [22], and to experience frustration and high levels of emotional distress when closeness with their partner was disrupted [84]: not only did they want more intimacy in their relationships, but they were also less likely to perceive intimacy [22,85], and they reported dissatisfaction in their ability to obtain the closeness they sought [86]. However, other studies [87], where attachment was defined as a dimension ranging from high comfort to high anxiety in a close relationship [88], showed only a weak relationship between level of intimacy and attachment. Some studies even found no significant effects of ambivalent or avoidant attachment styles on marital intimacy, but they did find a significant negative effect on partners’ commitment [89]. Furthermore, Constant and colleagues [90] argued that avoidantly attached people reported a poorer level of intra- and interpersonal emotional competences, as well as poorer intimacy in their romantic relationship; however, no associations between anxious attachment orientation and the sub-dimensions of intimacy (apart from engagement) were found. For women, joint regulation of emotions was additionally important in the context of intimacy, while intimacy for men involved the problem-focused and emotion-focused common DC of self and partner. Responding to a shared stressor by engaging in joint coping efforts facilitated feelings of trust, commitment, and closeness for both members of the couple, which enhanced intimacy [91].

Dyadic analysis confirmed that attachment-related avoidance was linked to lower intimacy and lower DC for women and men. Their own avoidant attachment predicted lower intimacy in men, but in women the effect was not significant. The partner’s attachment-related avoidance, however, predicted lower intimacy in men and women. These results are mostly consistent with theory and research, which suggest that the level of intimacy involved in a close relationship with a partner might be highly uncomfortable for individuals with high attachment avoidance [92]. These individuals generally have negative expectations of others, who they tend to expect to be unresponsive, unreliable, or unavailable to meet their needs [93]. They also require less time, affection, and self-disclosure to define a relationship as “close”, and not only want less intimacy but are also more sensitive to its presence, as compared with individuals who are less avoidant [22].

Our results indicate that spouses’ intimacy was affected by having children, and by their financial situation. Men with children reported lower intimacy, but in women this effect was not statistically significant. Simultaneously, the better the financial situation, the higher the level of intimacy in women, but not in men. Mothers’ actual or assumed primary responsibility for children means that they face greater family–work conflict compared to fathers [94]: their lower availability, and the fact that they devote less time to building relationships, may consequently result in men’s lower level of satisfaction with relational intimacy. Another possible interpretation is that in the modern family model, women are more concerned with their careers than in the traditional model [95], which may also translate into a reduced level of intimacy in men. The results of another study [70] indicate that Polish women more often communicate their needs and ask for help, whereupon men relieve women and take over their responsibilities, which seems to be a partial departure from the stereotypical model of a man, who is focused only on financial issues. On the other hand, women who are relieved of duties by their partners have a greater opportunity to build an intimate relationship with their partner. These results should be treated with caution, however, as most of the respondents (64.6%) did not have children, and only 4.1% of the couples had three or more children. An interesting result was the observation that, although the couple’s financial situation concerned both partners—especially as the majority of the surveyed couples were living together (97.5%)—it turns out that women more often assessed the financial situation of the family as bad (7.7%), compared to their partners. This may mean that as women become more involved in their work and careers, their sense of responsibility for family finances is also greater, as are their earnings expectations. As was confirmed in previous studies [96], this may also indicate a departure from the traditional and patriarchal family model, which is based on the roles of an independent, self-sufficient man and a dependent woman who expects the man to be responsible for the financial security of the family. At the same time, it can be assumed that the greater importance that women attach to the stable financial situation of the family may result from their greater need for financial security. A couple’s satisfaction with its financial status and financial decision-making are important for marital satisfaction [97]. Evolutionarily, this is consistent with women’s financial expectations in the context of family security [98,99] and financial security [100], which may translate into the higher overall satisfaction of women with their relationship [101], and their increased openness within it [102].

The above-mentioned issues concerning common areas of marital conflicts—i.e., division of childcare between partners, and family finances [103]—may refer to the concept of DC. Our results indicate that problem-focused common DC (but not emotion-focused common DC) moderates the effect of avoidant attachment on intimacy. Sharing positive and negative emotions with a partner gives a sense of acceptance, respect, and closeness [53], but individuals with high attachment avoidance generally feel less comfortable with offering or accepting support [61,104], and they report lower social and partner support [58,84]. Partners who feel more understood, appreciated, and cared for, in a situation of emotional communication, mitigate conflicts more easily, and become more involved in the relationship [105]. As sensitive care and support foster intimacy in a relationship, it is possible that support from a partner triggers attachment needs that are normally downplayed or denied by avoidant individuals, who would desire greater closeness with their partners when distressed, if they perceived their relationship to be high quality [106]. In our research, the avoidantly-attached were more inclined to accept instrumental rather than emotional support from a partner, which may not always be welcomed by avoidant individuals [107]. The partner’s inappropriate reaction may lead to these avoidant individuals distancing themselves [53,105]. However, accurate recognition of the partner’s needs and expectations may result in an avoidant individual’s greater openness to closeness and intimacy in the relationship [39].

In the present study, there were a number of limitations, such as its cross-sectional design and self-reported data. Additionally, our sample was relatively small (144 couples), 30% of the participants were couples in a short relationship (up to 5 years), and only 35% had at least one child. Gaps in the data prevented additional analyses based on children’s age, which can affect couples’ intimacy. Out of the 53 couples who had children, only 15 reported their age. Ten of the couples reported that their children were aged 4 years or less, while the remaining couples had children aged 17 to 23. In the APIM moderation analyses, our sample size may have limited our power to detect the possible moderation effects of emotion-focused DC. In addition, the results achieved in models 2 and 3, regardless of good fit indices, should be treated with caution.

Subsequent studies should focus on more homogeneous and representative groups, in terms of age and relationship duration, as this would allow more reliable results. In order to better understand the mechanisms of motivating avoidant partners to offer and receive support, in future research it would perhaps also be worth focusing on specific elements of DC, including the type of stress experienced by couples, its origin, and the sequence of its impact on individual partners [29]. There is definitely a need for more research, including longitudinal studies, into the relationship between DC and relationship functioning. Future research should also include interviews and observational data.

## 5. Conclusions

The conducted analyses led to the conclusion that although the relationship between partners’ avoidant attachment and their intimacy is strong, DC has the potential to promote the building of a close, trusting, and intimate relationship, despite attachment-related avoidance. The analyses not only made it possible to determine the significance of specific characteristics of the partners—in terms of attachment, intimacy, and DC—but also brought us closer to understanding the determinants of the partners’ intimacy in general, especially in the case of attachment-related avoidance. An undoubted advantage of the research was its dyadic character, and its ability to determine the importance of the mutual assessment of both partners in terms of the examined variables.

Finally, our study has clinical implications. Increasing intimacy and marital quality is often considered to be one of the most important goals in marital counselling [53]. Attachment insecurity—especially avoidant attachment—was found to be especially harmful to relationship outcome; therefore, treatment should be aimed at replacing insecure attachment strategies with the proximity-seeking secure primary attachment strategy [108,109]. Mutual support in a stressful situation can strengthen the bond between partners and develop empathy, commitment, sensitivity, and responsiveness to the partner’s needs. Competences that are important in the DC process translate into an increase in relational satisfaction and stability [110]; therefore, when working with couples, it is worth focusing on skills related to (a) mutual reading of emotions in stressful situations, (b) openness in expressing needs and fears, and (c) understanding and accepting the partner’s feelings [111]. Interventions related to reciprocal emotional exchange at the dyadic level usually improve the quality of interactions between partners [112], and also lead to an increase in the level of relational security, in which the partner is perceived as responsive and supportive, and the relationship is seen as lasting and satisfying [113]. DC can protect the relationship from the consequences of stress, strengthen the level of closeness and intimacy of partners, and increase the quality of the relationship [29,90].

## Figures and Tables

**Table 1:** Sample characteristics.

Variables	Female	Male
Gender	144 (50%)	144 (50%)
Age (M. SD)	31.9 (9.73)	30.88 (9.59)
Education		
Primary school	1 (0.7%)	1 (0.7%)
Middle school	18 (12.5%)	17 (11.9%)
Vocational school	1 (0.7%)	5 (3.4%)
Secondary school	57 (39.6%)	57 (39.9%)
High school	67 (46.5%)	63 (44,1%)
Employment status		
Full-time	102 (70.8%)	123 (89.8%)
Unemployed	8 (5.6%)	5 (3.6%)
Retired	1 (0.7%)	2 (1.5%)
Student	31 (21.5%)	7 (5.1%)
Maternity leave	2 (1.4%)	--------------
Financial situation		
Very good	26 (18%)	30 (21%)
Good	62 (43.1%)	59 (41,5%)
Rather good	45 (31.3%)	43 (30.3%)
Poor	11 (7.6%)	10 (7%)
Marital status		
Formal relationship	136 (47.2%)	
Informal relationship	152 (52.8%)	
Duration of the relationship		
1 to 5 years	144 (50.3%)	
6 to 10 years	64 (22.4%)	
11 to 20 years	36 (12.6%)	
21 to 30 years	20 (7%)	
Above 30 years	22 (7.7%)	
Children		
0	186 (64.8%)	
1	42 (14.6%)	
2	47 (16.4%)	
3 and more	12 (4.2%)	

**Table 2:** Descriptive statistics and probability for the Wilcoxon signed-rank test.

	Men				Women				*Z*
	*M*	*SD*	Skewness	Kurtosis	*M*	*SD*	Skewness	Kurtosis	
Security	43.5	7.14	−0.82	0.01	42.84	7.80	−0.47	−0.55	1.45
Ambivalence	21.64	8.6	0.66	−0.32	20.74	8.8	1.03	1.02	0.69
Avoidance	17.74	7.28	1.28	3.46	15.38	5.76	0.90	0.75	0.95
Intimacy	28.45	4.319	0.54	0.29	30.8	10.02	4.83	27.55 −0.49	−3.35 ***
Problem-focused CDC	12.17	2.55	−0.71	−0.17	12.31	2.32	−0.49	−0.49	−0.55
Emotion-focused CDC	7.20	2.05	−0.55	−0.11	7.33	2.06	−0.67	−0.09	−0.77

Note: *n* = 144 dyads; *** *p* < 0.001; CDC–common dyadic coping.

**Table 3:** Intercorrelations Among Path Model Variables for Women (_A) and Men (_P).

		1	2	3	4	5	6	7	8	9	10	11	12
1	Sec_A	1											
2	Amb_A	−0.04	1										
3	Avo_A	−0.40 **	0.16	1									
4	PCDC_A	0.38 **	−0.07	−0.24	1								
5	ECDC_A	0.34 **	−0.01	−0.14	0.51 **	1							
6	Intimacy_A	0.14	−0.02	−0.17	0.00	0.12	1						
7	Sec_P	0.64 **	0.01	−0.22 **	0.32 **	0.24 *	0.10	1					
8	Amb_P	0.03	**0.04**	0.10	−0.12	−0.06	−0.16	0.01	1				
9	Avo_P	−0.25 **	−0.12	0.18 *	−0.20 *	−0.22 **	−0.25 **	−0.23 **	0.17 *	1			
10	PCDC_P	0.32 **	0.09	−0.15	0.37 **	0.29 **	0.10	0.30 **	−0.06	−0.21 *	1		
11	ECDC_P	0.30 **	−0.01	−0.16	0.16	0.39 **	0.21 *	0.30 **	0.09	−0.13	0.44 **	1	
12	Intimacy_P	0.30 **	−0.05	−0.25 **	0.19 *	0.18 *	0.25 **	0.29 **	−0.10	−0.40 **	0.34 **	0.21 *	1

Note. *n* = 144 men and 144 women. Correlations between the dyad members are presented in bold along the diagonal. Sec: attachment security. Amb: ambivalent attachment. Avo: avoidant attachment. PCDC: problem-focused common DC. ECDC: emotion-focused common DC. * *p* < 0.05; ** *p* < 0.01.

**Table 4:** Actor and Partner effects of attachment-related avoidance on intimacy.

	Effect	Estimate	*p*	95% CI	Standardized Beta	r
Model 1	Women					
	Intercept	30.85	<0.001	29.28–32.42		
	Actor	−0.22	0.118	−0.50–0.06	−0.13	−0.13
	Partner	−0.40	0.005	−0.68–−0.12	−0.23	−0.23
	Men					
	Intercept	28.49	<0.001	27.86–29.12		
	Actor	−0.27	<0.001	−0.38–−0.16	−0.36	−0.37
	Partner	−0.14	0.015	−0.25–−0.03	−0.19	−0.20
Model 2	Women					
	Intercept	35.83	<0.001	30.61–41.05		
	Actor	−0.23	0.101	−0.50–0.05	−0.12	−0.13
	Partner	−0.40	0.005	−0.67–−0.12	−0.22	−0.23
	Men					
	Intercept	30.21	<0.001	28.12–32.30		
	Actor	−0.26	<0.001	−0.37–−0.15	−0.34	−0.37
	Partner	−0.14	0.013	−0.25–−0.03	−0.17	−0.20

**Table 5:** Effects in the problem-focused and emotion-focused common DC moderation model.

	Effect Type	Estimate	*p*	95% CI	Standardized Beta
Model 3					
Avoidance	Actor	−0.25	0.002	−0.40–0.09	−0.07
	Partner	−0.27	<0.001	−0.23–−0.14	−0.08
P_CDC	Actor	0.09	0.647	−0.29–0.47	0.06
	Partner	0.16	0.425	−0.23–0.54	0.11
Interaction	Actor–Actor	−0.03	0.069	−0.10–0.00	−0.08
	Actor–Partner	0.09	0.008	0.02–0.16	0.16
	Partner–Actor	−0.02	0.621	−0.09–0.05	−0.03
	Partner–Partner	−0.03	0.199	−0.08–−0.02	−0.06
Model 4					
Avoidance	Actor	−0.23	0.003	−0.38–0.09	−0.06
	Partner	−0.24	0.002	−0.06–−0.14	−0.06
E_CDC	Actor	0.15	0.525	−0.31–0.47	0.11
	Partner	0.39	0.092	−0.06–0.54	0.29
Interaction	Actor–Actor	−0.10	0.026	−0.18–0.00	−0.14
	Actor–Partner	0.42	0.424	−0.04–0.16	0.05
	Partner–Actor	0.48	0.481	−0.05–0.05	0.04
	Partner–Partner	−0.06	0.062	−0.16–−0.02	−0.12

P_CDC: problem-focused common DC. E_CDC: emotion-focused common DC.

## Data Availability

The data that support the findings of this study are available from the corresponding author upon reasonable request.
